# Normal limits of the electrocardiogram derived from a large database of Brazilian primary care patients

**DOI:** 10.1186/s12872-017-0572-8

**Published:** 2017-06-13

**Authors:** Daniel M. F. Palhares, Milena S. Marcolino, Thales M. M. Santos, José L. P. da Silva, Paulo R. Gomes, Leonardo B. Ribeiro, Peter W. Macfarlane, Antonio L. P. Ribeiro

**Affiliations:** 1 Telehealth Network of Minas Gerais, Hospital das Clínicas, Universidade Federal de Minas Gerais, Avenida Professor Alfredo Balena, 110, 1º Andar, Ala Sul, Sala 107, Belo Horizonte, 30130-100 Minas Gerais Brazil; 2 Medical School, Universidade Federal de Minas Gerais, Avenida Professor Alfredo Balena, 190, Belo Horizonte, 30130-100 Minas Gerais Brazil; 3 Institute of Cardiovascular and Medical Sciences, University of Glasgow, Glasgow, UK

**Keywords:** Electrocardiogram, Computers, Epidemiology, Intervals, Waves

## Abstract

**Background:**

Knowledge of the normal limits of the electrocardiogram (ECG) is mandatory for establishing which patients have abnormal ECGs. No studies have assessed the reference standards for a Latin American population. Our aim was to establish the normal ranges of the ECG for pediatric and adult Brazilian primary care patients.

**Methods:**

This retrospective observational study assessed all the consecutive 12-lead digital electrocardiograms of primary care patients at least 1 year old in Minas Gerais state, Brazil, recorded between 2010 and 2015. ECGs were excluded if there were technical problems, selected abnormalities were present or patients with selected self-declared comorbidities or on drug therapy. Only the first ECG from patients with multiple ECGs was accepted. The University of Glasgow ECG analysis program was used to automatically interpret the ECGs. For each variable, the 1st, 2nd, 50th, 98th and 99th percentiles were determined and results were compared to selected studies.

**Results:**

A total of 1,493,905 ECGs were recorded. 1,007,891 were excluded and 486.014 were analyzed. This large study provided normal values for heart rate, P, QRS and T frontal axis, P and QRS overall duration, PR and QT overall intervals and QTc corrected by Hodges, Bazett, Fridericia and Framingham formulae. Overall, the results were similar to those from other studies performed in different populations but there were differences in extreme ages and specific measurements.

**Conclusions:**

This study has provided reference values for Latinos of both sexes older than 1 year. Our results are comparable to studies performed in different populations.

## Background

The electrocardiogram (ECG) is a noninvasive, easy to perform, low cost test of wide clinical utility for investigation of the cardiac electrical activity with established diagnostic significance [1]. Knowledge of the normal ranges of measurements of intervals and axes of the pediatric and adult ECG is mandatory for establishing which patients have abnormal ECGs and who may therefore need special medical management. The use of computerized programs for automated ECG interpretation has shown good accuracy levels for ECG interval measurements, with benefits in saving time and money, and thus its use has increased for ECG analysis in epidemiological studies [2–5]. Different authors have studied digital ECGs using automated interpretation, and some have developed the reference values in different populations: Chinese, Caucasian, Blacks, South Asians and others [6–13].

Despite their importance, certain studies appear to have some gaps that require further investigation to complement current knowledge. For instance, some studies included only a small number of age groups or had small sample sizes for subjects of extreme ages or did not study reference values in children. Other studies did not contain all possible ECG variables. Of importance is the fact that there is lack of data regarding the normal limits specific to a Latin American population. Given the frequent immigration of this population into North America and Europe and the known ECG variations in different populations [14], the study of the normal limits of the ECG in Latinos is of importance for medical staff around the world.

Therefore, the aim of this study was to establish the normal limits of ECG measurements in apparently healthy Brazilians by using a large sample of pediatric and adult primary care patients in whom ECGs were recorded with a modern digital electrocardiograph. Measurements were obtained with an internationally known well-validated ECG program [5, 15]. In addition, the software had the capability to undertake Minnesota Coding [16] using automated techniques [17].

## Methods

### Study population

This retrospective observational study assessed all the 12-lead digital electrocardiograms of primary care patients of at least 1 year old in the state of Minas Gerais, Brazil, whose exams were sent to the Telehealth Network of Minas Gerais (TNMG) between 1st January 2010 and 21th January 2015. TNMG is a public telehealth service that was created in 2005 to provide support to the poorest cities of the state. After successive expansions, this service now assists the primary care professionals in 750 of the 853 cities. Minas Gerais is a special Brazilian state that can be considered representative in comparison to the rest of the country because of two main reasons. Firstly, while the north and northeast of Minas Gerais has HDI and poverty rates similar to the poorest states of Brazil, the south and central region of the state resembles the richest states of the country. Secondly, Minas Gerais is located in the middle of the country (southeast) and is the fourth largest state (586,521 Km^2^) with the second largest number of inhabitants in the country (20,869,101 [1, 18–20]).

ECGs were excluded from the study if any one of the following criteria was met:

- There was interference, artifacts or electrode placement errors (Minnesota Code [MC] 9.8.1 or 9.8.2);
- The ECG had an established abnormality: old myocardial infarction (major Q wave abnormalities [MC 1.1.x or 1.2.x]), possible old myocardial infarction (minor Q wave abnormalities plus ST-T abnormalities [MC 1.3 plus 4.1, 4.2, 5.1 or 5.2]), major isolated ST-T abnormalities (MC 4.1, 4.2, 5.1 or 5.2) complete or intermittent intraventricular blocks (MC 7.1, 7.2, 7.4 or 7.8), left ventricular hypertrophy plus ST-T abnormalities (MC 3.1 plus 4.1.x, 4.2, 5.1 or 5.2), major prolonged uncorrected QT interval (QTi ≥ 116%), major atrioventricular conduction abnormalities (MC 6.1, 6.2.x, 6.4, 6.8, 8.6.1 or 8.6.2), atrial fibrillation or flutter (MC 8–3-x), supraventricular tachycardia (MC 8.4.2 or 8–4-1 with heart rate > 140 bpm), wandering atrial pacemaker (MC 8.1.4), supraventricular rhythm persistent (MC 8.4.1 plus heart rate ≤ 140), high amplitude P wave (MC 9.3), asystole or ventricular fibrillation (MC 8.2);
- Repeated exams: for the purpose of this analysis, only the first ECG from patients with multiple ECGs was analyzed.
- Patient had these self-declared comorbidities or cardiovascular risk factors: arterial hypertension, diabetes, smoking, dyslipidemia, personal history of myocardial infarction, personal history of coronary revascularization, Chagas disease and chronic pulmonary disease;
- Patient was receiving any kind of drug therapy (diuretics, digitalis, beta-blockers, angiotensin-converting-enzyme inhibitors, amiodarone, calcium channel blockers or any drug listed in the “others” field).

The study population was divided into 14 age groups: 01–02, 03–04, 05–07, 08–11, 12–15, 16–19, 20–29, 30–39, 40–49, 50–59, 60–69, 70–79, 80–89 years, and 90 years and older.

### Data acquisition

Each primary care center received one of the two available digital electrocardiographs to record the digital 12-lead ECGs, ErgoPC 13 (MICROMED, Brazil) or ECG PC (TEB, Brazil), and one specific software that allowed entry of clinical information based on patient’s self-report and local medical record. The data acquisition rate of this two devices were 500 Hz and 600 Hz at 5 and 3.9 μV resolution, respectively. Thereafter, the ECGs with all personal information attached, were sent through the internet to the analysis center of the TNMG and were saved in a database that was used for data collection in the present study. All the patients’ data were collected by the primary care practitioners and represent self-declared information.

### Database

For each patient, the database contains:

- patient identification: full name, gender, birth date and city of domicile;
- comorbidities and cardiovascular risk factors: arterial hypertension, obesity, diabetes, smoking, dyslipidemia, personal history of myocardial infarction, personal history of coronary revascularization, family history of coronary disease, Chagas disease and chronic pulmonary disease;
- medications: diuretics, digitalis, beta-blockers, angiotensin-converting-enzyme inhibitors, amiodarone and calcium channel blockers) or free text field (“others”);
- symptoms: chest pain and any equivalent;
- anthropometric measures: height and weight;
- 12 lead ECG recordings;
- administrative information about the ECG: date and city in which it was performed.

Patients’ information (name, birth date and city) was standardized to avoid errors in identification, which could cause the inclusion of the same patient twice. Due to the frequent miscegenation in the Brazilian population, data from race was not considered in this study. Also, other information like pregnancy was not available and the healthcare professional could not fill out the form with blood pressure at the time the ECG was recorded.

### The Glasgow program

The University of Glasgow (Uni-G) ECG analysis program (release 28.5, issued on January 2014) is internationally well-recognized computer software that was used to automatically interpret the ECGs in the TNMG database [3]. It has been in continuous development for over 25 years and it is applicable to neonates as well as adults [5, 15]. This program has been extensively evaluated, meeting the requirements of IEC 60601–2-25 and is used routinely world-wide. It provides all the standard amplitude, duration and axes measurements as well as a rhythm analysis and diagnostic interpretation [21–23]. It is well suited for epidemiological studies [3–5, 15].

The Uni-G program uses strict standards of electrocardiographic interpretation and allows the export of two types of diagnostic statements, long and short:

□ Quantitative description: average heart rate; P, QRS, and T axes; P and QRS durations; PR and QT intervals; and corrected QT (QTc) by the methods of Framingham, Hodges, Bazett and Fridericia;

□ Qualitative description: the software uses the quantitative description to automatically classify the electrocardiographic abnormalities according to the Minnesota Code.

**Fig. 1:**
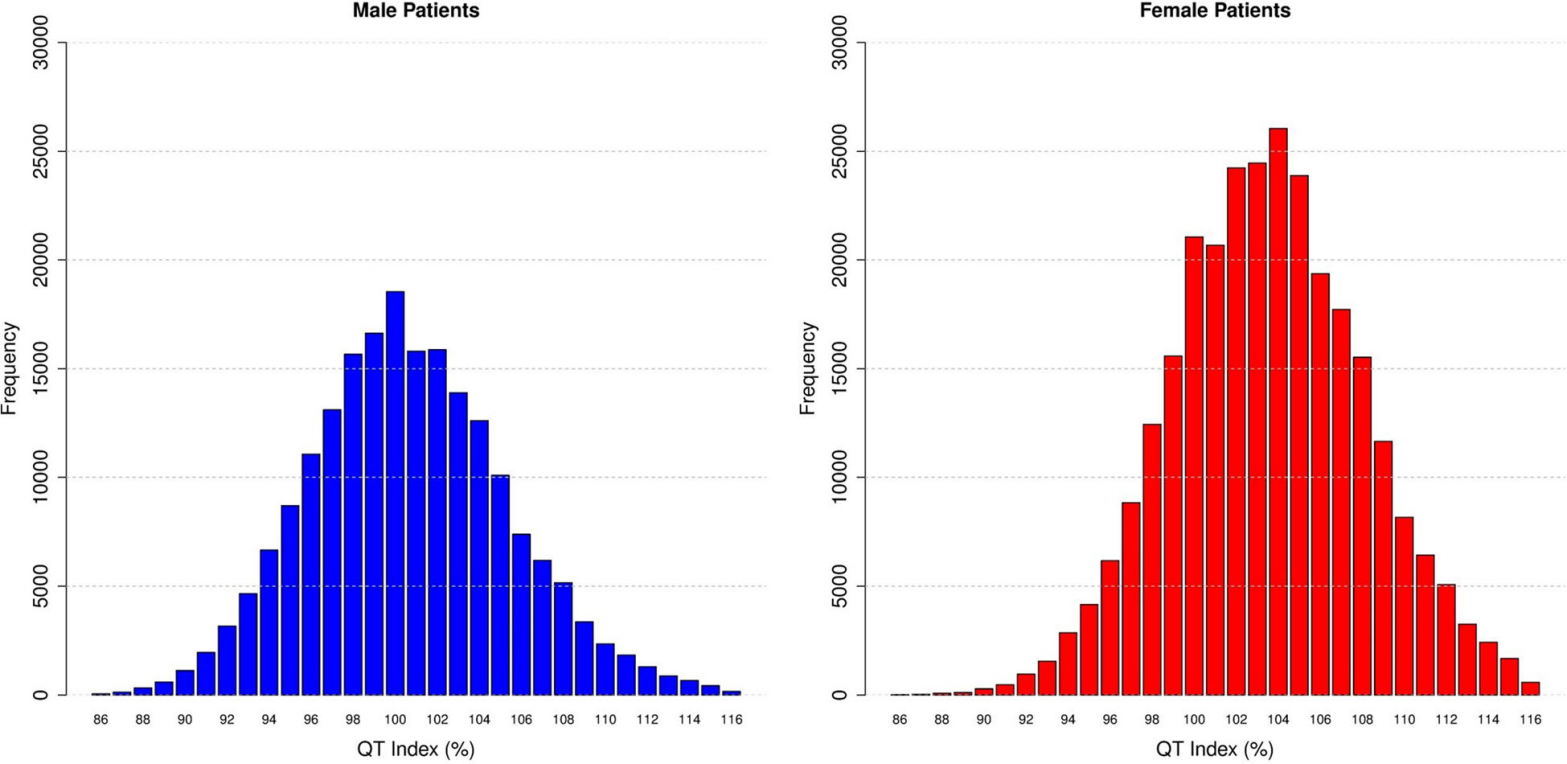
QT index according sex

All the ECGs had their quantitative data analyzed, and reference values were established for each variable for the study population. QT index (QTi) was calculated according the formulae QTi = (QT / 656) x (heart rate + 100) [24, 25] and its prevalence was demonstrated in Fig. 1.

### Comparison with other studies

A systematic review of available literature was undertaken to identify relevant publications in English about electrocardiographic reference values for heart rate, axis and measurements that were derived from digital ECGs and a sample size of at least one thousand patients. A comprehensive search was conducted in the electronic database PubMed (covering all dates up to July 30, 2015), using the following keywords and their combinations: “digital”, “electrocardiogram”, “electrocardiographic”, “ecg”, “ekg”, “reference values”, “reference ranges”, “normal limits”, “normal values” and “epidemiology”. Additional strategies included reviews of journals or periodicals not indexed in the above mentioned electronic database. From all the available studies, we chose four that included patients with age groups, percentiles and variables similar to those that were included in the present study and we compared all the results according age and sex based groups [7, 12, 13, 26]. Only one of them included data for percentiles 1 and 99 [13], but all included percentiles 2, 50 and 98 (Table 1). Because not all studies included all of the variables which we studied, some comparisons could not be undertaken among all the studies. Different age groups were not compared, except when we considered that the difference was irrelevant eg. 90-99 y versus age ≥ 90 y.

**Table 1:** Selected studies of normal ECG intervals

Study	Population	Year	Patients (n)	ECG acquisition	Sampling rate	Analytic program	Percentiles
Rijnbeek et al.	0 to 16 y; Dutch	2001	Male (944); Female (968)	Cardio Control (Delft, Netherlands)	1200 Hz	MEANS^a^	2nd, 50th and 98th
Wu et al.	18 to ≥ 60 y; Chinesese	2003	Male (3614); Female (1746)	Cardio Control (Delft, Netherlands)	1200 Hz	MEANS^a^	2nd, 50th and 98th
Mason et al.	0 to 99 years; Northern America (70%), Europe (21%), Africa (3%), Latin America (3%), and Asia (2%) and Oceania (2%).	2007	Male (14,297); Female (12,201)	MTX-2 (CCSSI); MAC 1200 (GE Medical Systems)	500 Hz	CCSSI	1st, 2nd, 50th, 98th, 99th
Rijnbeek et al.	16 to 89 y; Dutch	2014	Male (7326); Female (6028)	ACTA (ESAOTE, Florence, Italy); Cardio Perfect equipment (Welch Allyn Cardio Control, USA); Megacart (Siemens, Erlangen, Germany)	500 Hz	MEANS^a^	2nd, 50th and 98th

^a^Modular ECG Analysis System (MEANS)

### Statistical analysis and estimation of normal values

Descriptive statistics were computed for the whole database. Categorical data were reported as counts and percentages; continuous variables were reported as mean and standard deviation or median and percentiles, as appropriate. For each ECG variable, the median plus the 1st, 2nd, 98th and 99th percentiles of the measurement distribution per age and gender were determined. The 2nd percentile was taken as the lower limit and the 98th percentile as the upper limit of the normal range.

For analysis purposes, age was rounded to the nearest integer. Then, for each integer age, the reference percentile of the quantitative ECG variable was computed. Subsequently, the locally weighted polynomial regression method (LOESS) was used to graphically display a smoothed relationship for this reference percentile as a function of age for patients from 1 to 90 years old [27]. The span (smoothing) parameter was chosen after a visual inspection and was set to 0.40.

Graphs with the percentiles 2nd, 50th and 98th for each age group and both sexes were created to compare the result of different studies.

Data management and statistical computations were performed with IBM SPSS Statistics for Windows version 20.0 (IBM Corp. Released 2011. Armonk, NY: IBM Corp) and R statistical computing software version 3.2.0 with *foreign* and *plyr* packages. Loess curves were calculated using the R *loess* function [27].

This study was approved by the Research Ethics Committee of the Universidade Federal de Minas Gerais.

## Results

### Characteristics of the study population

**Fig. 2:**
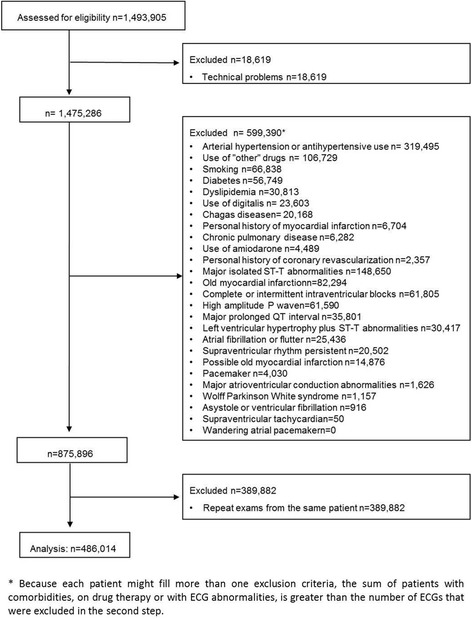
Excluded patients

**Fig. 3:**
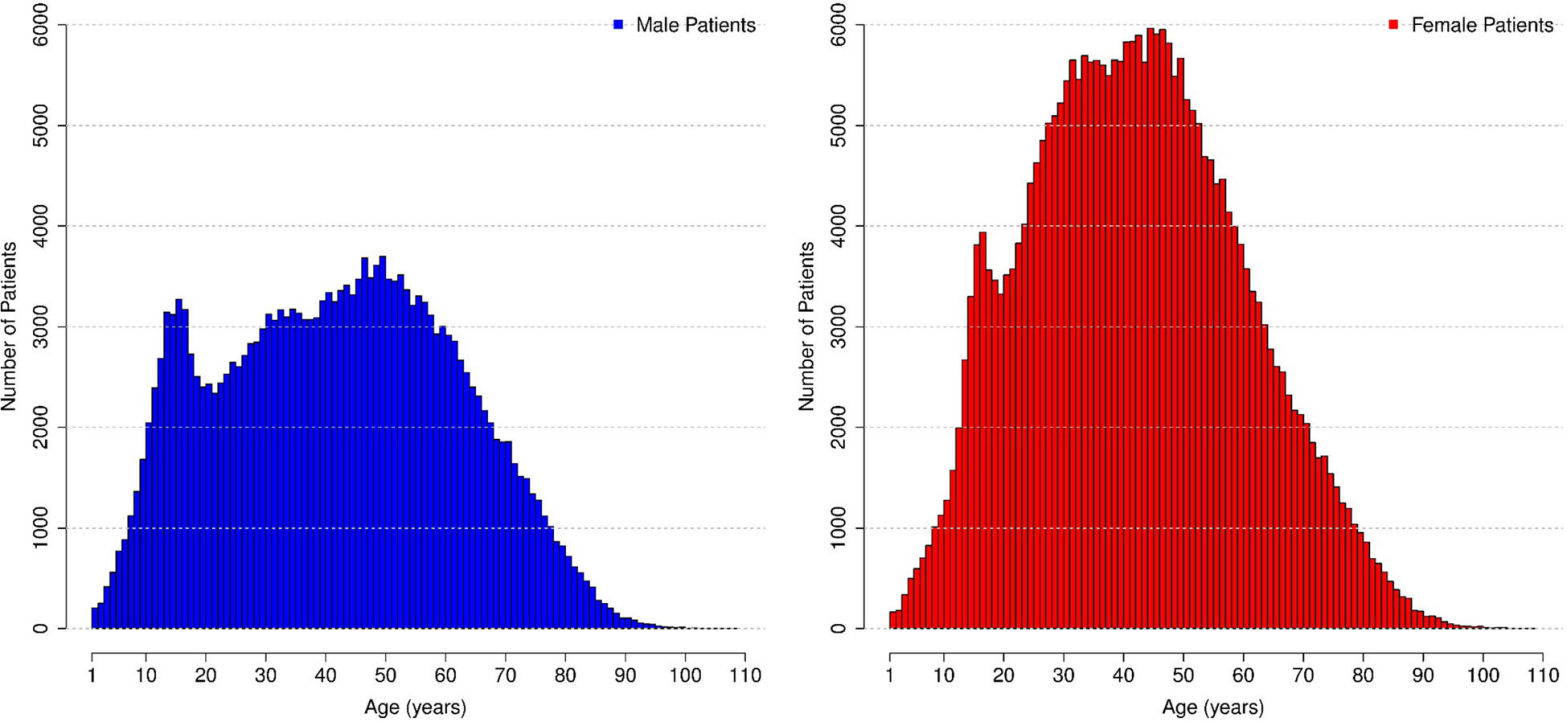
Age and sex distribution of the study population

A total of 1,493,905 ECGs were recorded during the study period. The exclusion criteria were applied in consecutive steps (Fig. 2). First, 18,619 ECGs with technical problems were excluded. From the 1,475,286 remaining ECGs, a total of 599,390 patients on various medicines or with selected ECG abnormalities or self-declared comorbidities were excluded. Finally, 389,882 ECGs from patients who had more than one ECG recorded during study period were ruled out. After all the exclusion criteria were applied, 486,014 ECGs were analyzed (patients’ mean age 42.2 ± 18.6 years, 58.8% females). Figure 3 and Table 2 describe the study population.

**Table 2:** Age and sex distribution of the study population

Age group (years)	Male	Female	Total	
	n	n	n	%
1–2	259	216	475	0.1%
3–4	840	667	1507	0.3%
5–7	2486	1947	4433	0.9%
8–11	6802	4601	11,403	2.4%
12–15	11,812	10,709	22,521	4.6%
16–19	11,295	14,453	25,748	5.3%
20–29	26,063	43,281	69,344	14.3%
30–39	31,138	55,671	86,809	17.9%
40–49	34,414	57,942	92,356	19.0%
50–59	32,988	46,563	79,551	16.4%
60–69	24,149	28,620	52,769	10.9%
70–79	13,499	15,280	28,779	5.9%
80–89	4120	4998	9118	1.9%
> = 90	482	719	1201	0.2%
Total	200,347	285,667	486,014	100%

### Electrocardiographic findings

#### Global measurements

Tables 3 and 4 show percentiles 1st,2nd, 50th, 98th and 99th for heart rate, P, QRS and T frontal axis, overall P and QRS duration, overall P and QT interval and QTc corrected by Hodges, Bazett, Fridericia and Framingham methods for different age groups and both sexes. All global parameters showed age trends and sex differences [28–31].

**Table 3:** ECG parameters for patients from 1 to 29 years-old: (1st percentile; 2nd percentile; 50th percentile; 98th percentile; 99th percentile)

Parameter	Sex	1–2	3–4	5–7	8–11	12–15	16–19	20–29
Heart rate (bpm)	Male	(52; 59; 107; 166; 170)	(67; 70; 97; 132; 139)	(59; 61; 86; 120; 126)	(53; 55; 76; 108; 114)	(47; 49; 69; 101; 107)	(44; 46; 65; 98; 105)	(44; 46; 65; 98; 104)
	Female	(58; 66; 108; 163; 199)	(64; 69; 98; 138; 141)	(62; 64; 88; 124; 133)	(56; 58; 81; 115; 121)	(52; 54; 75; 107; 114)	(51; 53; 73; 105; 112)	(51; 53; 73; 104; 111)
P Frontal Axis (°)	Male	(−95; −10; 53; 85; 116)	(−30; −14; 50; 79; 96)	(−34; −22; 44; 74; 81)	(−35; −21; 41; 74; 79)	(−33; −20; 44; 77; 82)	(−32; −21; 54; 81; 85)	(−22; −13; 54; 80; 85)
	Female	(−12; −6; 53; 78; 85)	(−30; −10; 51; 76; 90)	(−19; −11; 49; 74; 83)	(−21; −12; 45; 74; 78)	(−21; −13; 48; 76; 80)	(−22; −13; 51; 77; 82)	(−18; −11; 51; 78; 81)
QRS Frontal Axis (°)	Male	(−46; −26; 61; 124; 132)	(−21; −9; 61; 99; 111)	(−29; −10; 64; 101; 111)	(−31; −8; 64; 99; 107)	(−28; −11; 66; 101; 107)	(−41; −18; 68; 100; 107)	(−37; −20; 59; 96; 101)
	Female	(−49; −20; 62; 102; 136)	(−22; −3; 66; 102; 108)	(−9; 0; 67; 100; 109)	(−14; 0; 66; 98; 104)	(−8; 2; 65; 95; 100)	(−13; 0; 63; 95; 99)	(−16; −5; 56; 89; 95)
T Frontal Axis (°)	Male	(−9; 2; 43; 70; 73)	(5; 9; 43; 65; 69)	(7; 13; 45; 67; 70)	(7; 12; 46; 68; 71)	(2; 9; 48; 71; 73)	(−3; 3; 47; 72; 75)	(−12; −5; 40; 71; 75)
	Female	(8; 12; 43; 73; 77)	(−4; 0; 42; 68; 70)	(4; 9; 44; 68; 71)	(1; 8; 45; 69; 72)	(0; 4; 44; 69; 73)	(−2; 3; 42; 70; 73)	(−7; −1; 38; 68; 72)
Overall P Duration (ms)	Male	(63; 64; 84; 124; 142)	(64; 70; 86; 112; 124)	(64; 68; 88; 114; 128)	(68; 72; 94; 118; 132)	(68; 72; 98; 124; 134)	(68; 74; 102; 126; 134)	(72; 78; 106; 132; 138)
	Female	(52; 63; 84; 115; 128)	(64; 68; 86; 115; 127)	(68; 72; 90; 116; 128)	(70; 72; 94; 116; 130)	(72; 76; 98; 124; 132)	(72; 78; 100; 128; 134)	(74; 78; 104; 132; 138)
Overall PR Interval (ms)	Male	(81; 86; 118; 184; 192)	(90; 92; 122; 170; 186)	(90; 94; 124; 174; 188)	(96; 100; 132; 180; 190)	(98; 104; 136; 188; 202)	(102; 106; 142; 196; 206)	(104; 110; 148; 200; 212)
	Female	(84; 87; 118; 171; 178)	(88; 90; 120; 166; 171)	(90; 94; 124; 166; 173)	(94; 98; 128; 178; 188)	(98; 102; 136; 184; 196)	(98; 104; 138; 188; 198)	(102; 106; 142; 192; 202)
Overall QRS Duration (ms)	Male	(60; 62; 74; 98; 101)	(64; 66; 76; 92; 96)	(66; 66; 80; 99; 102)	(68; 70; 84; 102; 106)	(72; 74; 90; 108; 112)	(74; 76; 94; 114; 116)	(76; 78; 96; 114; 116)
	Female	(58; 60; 72; 97; 100)	(62; 62; 74; 90; 94)	(62; 64; 78; 96; 98)	(66; 68; 80; 98; 100)	(68; 70; 84; 102; 106)	(70; 70; 86; 104; 108)	(70; 72; 88; 106; 110)
QT Interval (ms)	Male	(239; 250; 314; 412; 426)	(276; 281; 332; 396; 405)	(294; 300; 356; 412; 423)	(314; 322; 378; 436; 446)	(324; 332; 390; 452; 460)	(320; 328; 390; 454; 462)	(324; 332; 390; 456; 464)
	Female	(224; 255; 314; 419; 454)	(271; 278; 332; 396; 412)	(286; 296; 350; 402; 408)	(306; 314; 368; 426; 434)	(314; 322; 380; 440; 450)	(316; 326; 382; 442; 450)	(320; 328; 386; 446; 454)
QTc Hodges (ms)	Male	(367; 370; 405; 444; 455)	(364; 369; 399; 430; 437)	(370; 375; 403; 436; 441)	(374; 377; 408; 440; 445)	(370; 374; 408; 446; 452)	(364; 368; 400; 442; 450)	(365; 368; 400; 442; 449)
	Female	(363; 367; 404; 459; 470)	(369; 371; 401; 436; 440)	(371; 375; 403; 431; 437)	(373; 378; 407; 438; 442)	(375; 379; 409; 443; 449)	(373; 377; 408; 443; 449)	(375; 379; 411; 447; 452)
QTc Bazett (ms)	Male	(377; 384; 421; 459; 469)	(377; 384; 421; 460; 467)	(381; 388; 425; 463; 469)	(381; 386; 425; 463; 469)	(368; 374; 419; 462; 468)	(358; 364; 406; 452; 458)	(359; 364; 406; 451; 459)
	Female	(380; 383; 421; 467; 479)	(382; 388; 424; 464; 470)	(385; 390; 425; 461; 467)	(384; 389; 427; 465; 471)	(382; 387; 426; 466; 471)	(377; 383; 423; 463; 469)	(379; 385; 426; 466; 471)
QTc Fridericia (ms)	Male	(332; 342; 381; 429; 434)	(349; 358; 389; 427; 432)	(361; 367; 400; 439; 443)	(371; 376; 408; 442; 447)	(368; 372; 408; 447; 453)	(362; 366; 400; 441; 448)	(363; 367; 400; 439; 446)
	Female	(332; 339; 383; 445; 453)	(354; 358; 391; 431; 438)	(361; 366; 398; 433; 436)	(369; 374; 406; 440; 444)	(372; 377; 410; 446; 451)	(371; 375; 408; 444; 450)	(374; 378; 411; 448; 454)
QTc Framingham (ms)	Male	(336; 344; 382; 429; 435)	(355; 361; 390; 426; 430)	(364; 371; 401; 436; 441)	(374; 378; 409; 442; 446)	(368; 373; 409; 446; 452)	(359; 365; 400; 440; 447)	(361; 365; 400; 438; 444)
	Female	(332; 345; 382; 441; 448)	(358; 359; 390; 428; 435)	(363; 369; 399; 430; 434)	(372; 376; 406; 439; 444)	(375; 379; 410; 444; 450)	(373; 377; 409; 444; 449)	(375; 380; 412; 447; 453)

**Table 4:** ECG parameters for patients older than 30 years-old: (1st percentile; 2nd percentile; 50th percentile; 98th percentile; 99th percentile)

Parameter	Sex	30–39	40–49	50–59	60–69	70–79	80–89	> = 90
Heart rate (bpm)	Male	(44; 46; 65; 99; 105)	(44; 46; 66; 98; 104)	(44; 46; 66; 98; 104)	(44; 46; 66; 99; 104)	(45; 47; 66; 99; 105)	(45; 47; 68; 104; 111)	(47; 49; 70; 109; 118)
	Female	(50; 52; 72; 102; 107)	(49; 52; 71; 101; 107)	(48; 50; 70; 99; 105)	(48; 50; 70; 101; 106)	(48; 50; 71; 102; 106)	(48; 51; 73; 105; 111)	(50; 52; 76; 111; 120)
P Frontal Axis (°)	Male	(−15; −8; 53; 80; 85)	(−13; −5; 55; 80; 85)	(−12; −3; 57; 82; 87)	(−11; −3; 58; 84; 93)	(−12; −5; 60; 86; 95)	(−25; −9; 61; 94; 103)	(−35; −23; 61; 95; 101)
	Female	(−15; −8; 52; 78; 82)	(−13; −5; 53; 79; 83)	(−12; −4; 53; 79; 83)	(−12; −5; 54; 80; 85)	(−13; −5; 57; 84; 94)	(−15; −7; 58; 85; 97)	(−45; −13; 59; 85; 99)
QRS Frontal Axis (°)	Male	(−41; −28; 47; 90; 96)	(−48; −36; 38; 85; 91)	(−56; −45; 31; 82; 85)	(−61; −51; 23; 80; 84)	(−65; −57; 18; 79; 84)	(−70; −62; 15; 78; 83)	(−69; −62; 15; 84; 98)
	Female	(−24; −14; 46; 84; 87)	(−32; −23; 37; 80; 85)	(−41; −33; 28; 77; 82)	(−48; −40; 21; 74; 80)	(−53; −46; 17; 73; 79)	(−58; −51; 15; 72; 78)	(−63; −55; 13; 77; 80)
T Frontal Axis (°)	Male	(−18; −10; 35; 71; 75)	(−20; −12; 35; 73; 77)	(−23; −13; 39; 77; 83)	(−26; −15; 43; 81; 88)	(−27; −15; 48; 85; 95)	(−28; −18; 52; 90; 102)	(−37; −14; 55; 95; 99)
	Female	(−12; −4; 36; 69; 72)	(−16; −8; 36; 72; 76)	(−18; −9; 38; 77; 83)	(−18; −8; 42; 81; 90)	(−21; −10; 46; 85; 95)	(−23; −12; 50; 89; 99)	(−26; −11; 52; 97; 104)
Overall P Duration (ms)	Male	(76; 82; 110; 136; 140)	(78; 84; 110; 136; 140)	(78; 84; 112; 138; 144)	(74; 82; 114; 140; 146)	(72; 78; 114; 142; 146)	(68; 74; 114; 146; 150)	(62; 72; 114; 146; 150)
	Female	(74; 80; 106; 134; 138)	(76; 80; 106; 136; 140)	(76; 82; 108; 136; 142)	(76; 80; 110; 138; 144)	(72; 78; 112; 142; 146)	(68; 74; 112; 144; 150)	(68; 72; 112; 144; 148)
Overall PR Interval (ms)	Male	(108; 112; 150; 204; 216)	(108; 112; 152; 204; 216)	(106; 114; 152; 208; 220)	(108; 114; 154; 216; 228)	(108; 114; 158; 230; 246)	(108; 114; 164; 246; 268)	(106; 115; 166; 253; 289)
	Female	(102; 106; 144; 194; 206)	(102; 106; 144; 194; 204)	(104; 110; 148; 200; 210)	(104; 110; 150; 208; 218)	(106; 110; 154; 214; 230)	(106; 112; 156; 232; 244)	(106; 108; 160; 241; 251)
Overall QRS Duration (ms)	Male	(76; 78; 96; 114; 116)	(76; 78; 94; 114; 116)	(74; 76; 94; 114; 116)	(74; 76; 94; 114; 116)	(74; 76; 94; 114; 116)	(72; 76; 94; 116; 116)	(70; 72; 92; 116; 118)
	Female	(70; 72; 88; 108; 110)	(72; 72; 88; 108; 112)	(72; 74; 90; 110; 112)	(72; 74; 90; 110; 114)	(70; 72; 90; 112; 114)	(70; 72; 88; 112; 116)	(66; 70; 88; 110; 112)
QT Interval (ms)	Male	(324; 334; 392; 458; 468)	(328; 336; 396; 460; 472)	(328; 336; 398; 466; 478)	(328; 338; 402; 472; 484)	(326; 336; 404; 474; 486)	(320; 332; 406; 476; 488)	(307; 319; 400; 480; 491)
	Female	(326; 334; 392; 452; 460)	(328; 338; 396; 458; 468)	(332; 340; 402; 466; 474)	(330; 338; 402; 470; 480)	(328; 338; 402; 470; 478)	(320; 332; 400; 470; 480)	(309; 319; 394; 473; 480)
QTc Hodges (ms)	Male	(368; 372; 404; 445; 451)	(371; 375; 407; 450; 456)	(374; 378; 411; 454; 461)	(375; 380; 414; 459; 466)	(377; 382; 417; 462; 468)	(378; 383; 421; 465; 470)	(380; 385; 420; 471; 478)
	Female	(379; 383; 414; 452; 458)	(381; 385; 418; 456; 462)	(382; 387; 421; 460; 466)	(383; 388; 423; 464; 470)	(383; 388; 424; 464; 469)	(382; 388; 425; 465; 470)	(381; 385; 423; 465; 470)
QTc Bazett (ms)	Male	(363; 369; 411; 457; 463)	(367; 372; 414; 462; 468)	(370; 376; 418; 465; 471)	(373; 379; 421; 468; 474)	(375; 382; 426; 472; 478)	(379; 385; 431; 475; 479)	(375; 386; 434; 479; 484)
	Female	(382; 388; 429; 470; 476)	(385; 391; 432; 473; 478)	(386; 392; 435; 475; 480)	(386; 393; 437; 476; 481)	(389; 395; 439; 478; 481)	(389; 396; 441; 480; 483)	(383; 397; 442; 483; 485)
QTc Fridericia (ms)	Male	(367; 371; 404; 443; 449)	(370; 374; 407; 448; 454)	(372; 377; 411; 452; 458)	(374; 378; 414; 456; 461)	(374; 380; 418; 461; 465)	(376; 381; 421; 463; 467)	(377; 380; 420; 471; 473)
	Female	(377; 382; 416; 454; 459)	(379; 384; 419; 458; 463)	(381; 386; 423; 461; 465)	(381; 387; 425; 463; 468)	(380; 386; 426; 464; 467)	(377; 384; 427; 465; 469)	(371; 382; 425; 468; 470)
QTc Framingham (ms)	Male	(365; 370; 404; 442; 448)	(368; 373; 407; 447; 453)	(371; 376; 410; 451; 457)	(373; 378; 414; 455; 460)	(375; 380; 417; 460; 464)	(376; 382; 420; 462; 466)	(375; 380; 420; 470; 473)
	Female	(379; 383; 416; 453; 458)	(381; 386; 419; 457; 462)	(382; 387; 423; 460; 464)	(383; 388; 424; 463; 467)	(381; 388; 426; 463; 467)	(380; 386; 426; 465; 468)	(375; 381; 424; 465; 469)

#### Heart rate

**Fig. 4:**
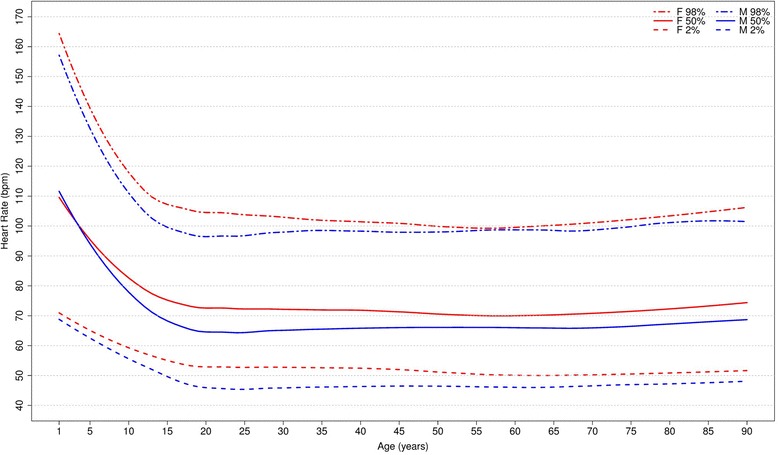
Percentiles 2nd, 50th and 98th for heart rate according age and sex

Figure 4 shows our reference values for heart rate. In males, it decreases during childhood and adolescence, reaches a median value of 65 bpm by the age of 16, stabilizes around 65–66 bpm from 20 to 79 years and increases to 70 bpm after 90 years-old. In females, the heart seems to beat faster. It also decreases during the first part of life, but reaches a median value of 73 bpm at age 16, then fluctuates around 70 to 73 bpm until 79 years old and increases to 76 bpm in women older than 90 years-old.

**Fig. 5:**
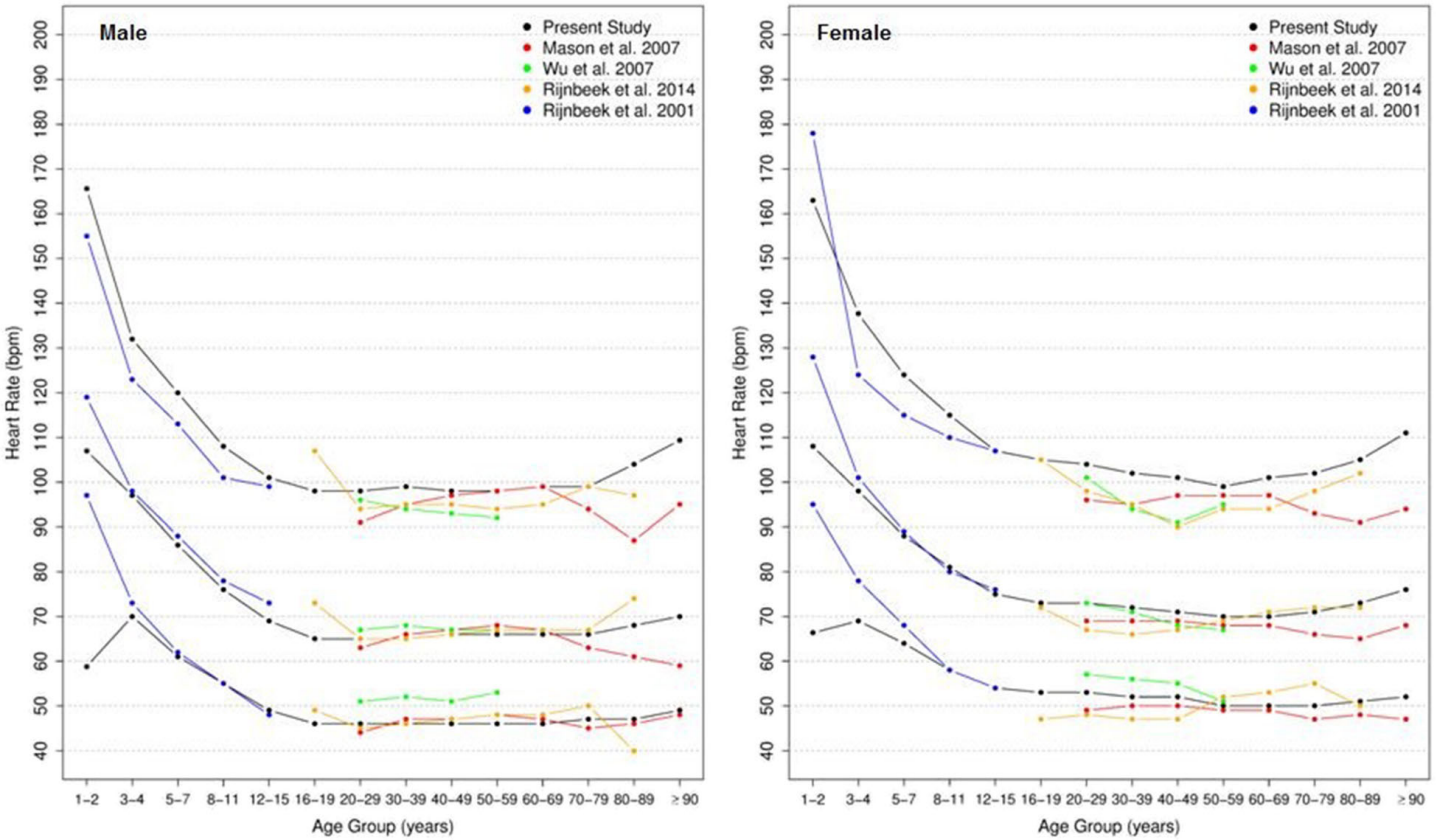
Comparison of lower, median and upper normal limits for heart rate of different studies according age groups and sex

The percentiles for heart rate were compared to those of Mason [13], Wu [7] and Rijnbeek [12, 26] studies (Fig. 5), and we considered that our results were similar for most age groups in both sexes. However, some major differences were seen in results for men from 1-2 y, 16-19 y, 80-89 y and ≥90 y, and women from 1-2y, 3-4 y, 80-89 y and ≥90 y.

#### P, QRS and T frontal axis

**Fig. 6:**
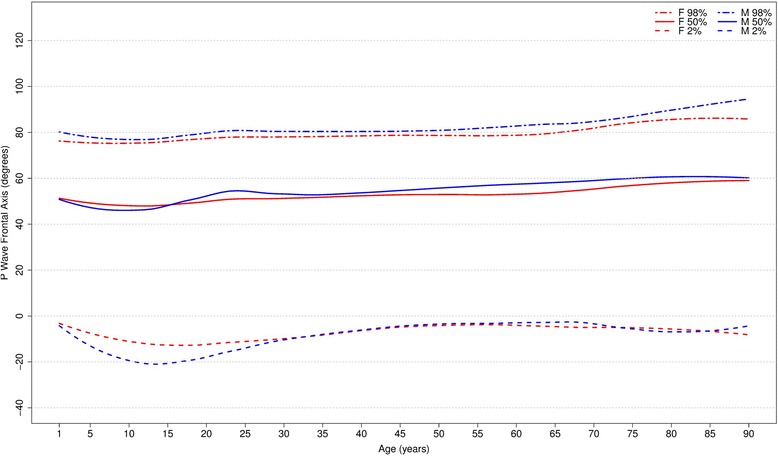
Percentiles 2nd, 50th and 98th for P-wave frontal axis according age and sex

**Fig. 7:**
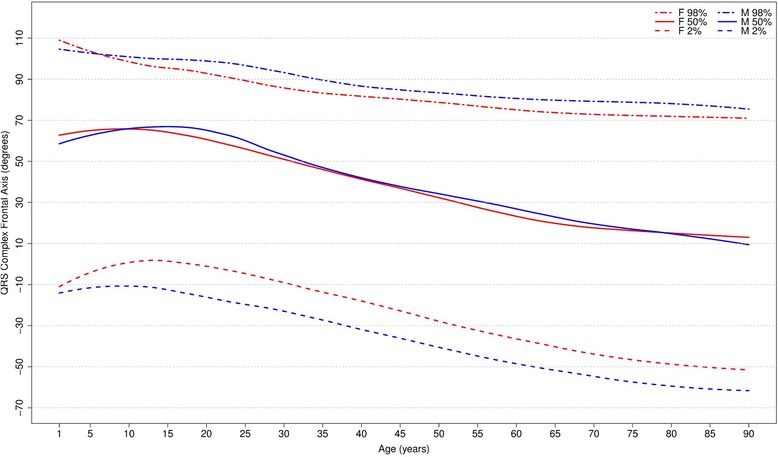
Percentiles 2nd, 50th and 98th for QRS-wave frontal axis according age and sex

**Fig. 8:**
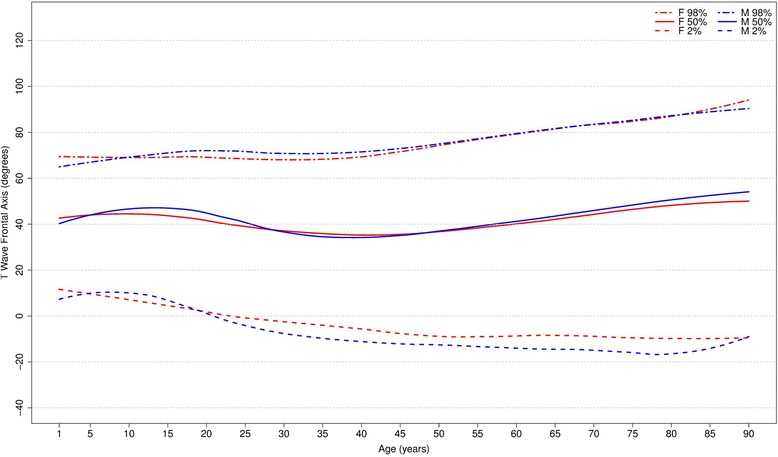
Percentiles 2nd, 50th and 98th for T-wave frontal axis according age and sex

Figures 6, 7 and 8 show our results for the normal limits of the P, QRS and T-wave frontal axes, respectively.

With respect to the P wave, the male P axis is generally, after 8 years of age, slightly more orientated in the inferior direction compared to that of females. For both males and females, the median P-wave frontal axis shifts superiorly during childhood, until 8–11 years, and then turns inferiorly with advancing age. Notwithstanding these small changes, the P wave axis is relatively constant throughout life.

It is well known that the median QRS axis shifts counterclockwise in the neonate and infant in the weeks and months after birth [32]. In our study, where the youngest participant was aged 1 year, the median QRS wave frontal axis shifted inferiorly for both sexes during childhood and by the age of 12–15 for females and 16–19 for males, it shifted superiorly, reaching 15 and 13 degrees for men and women, respectively, after 90 years-old. Although the median value is very similar for both sexes, men have a wider reference range during the whole of life.

The T wave frontal axis is very similar for men and women. The median varies from 43 to 38 and 43 to 45 degrees for men and women, respectively, until 20 years of age. In adult life, it rotates superiorly to 35–40 degrees but then moves inferiorly after 50 years of age for both sexes. The upper and lower limits tend to diverge progressively with aging.

**Fig. 9:**
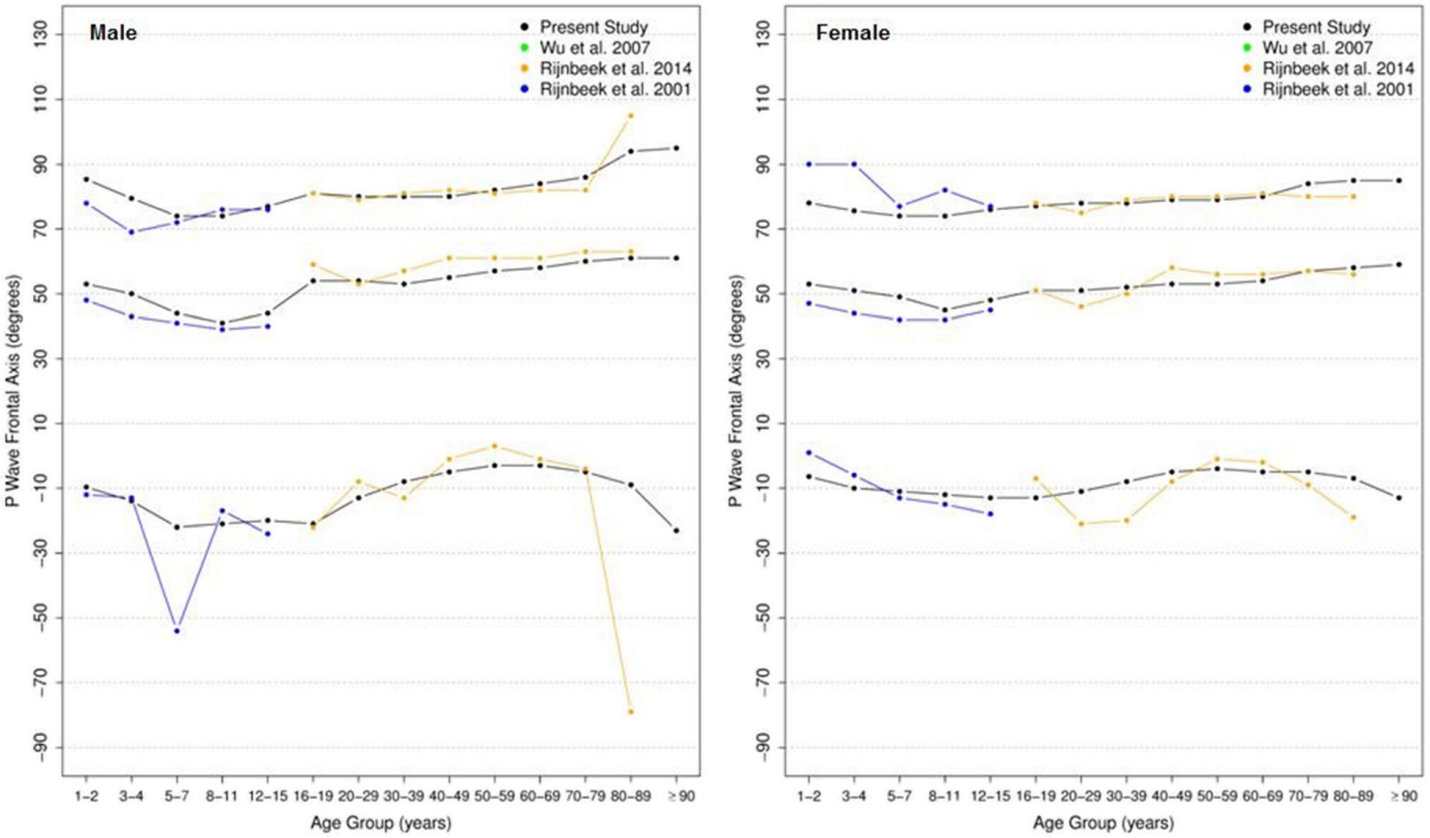
Comparison of lower, median and upper normal limits for P-wave frontal axis of different studies according age groups and sex

**Fig. 10:**
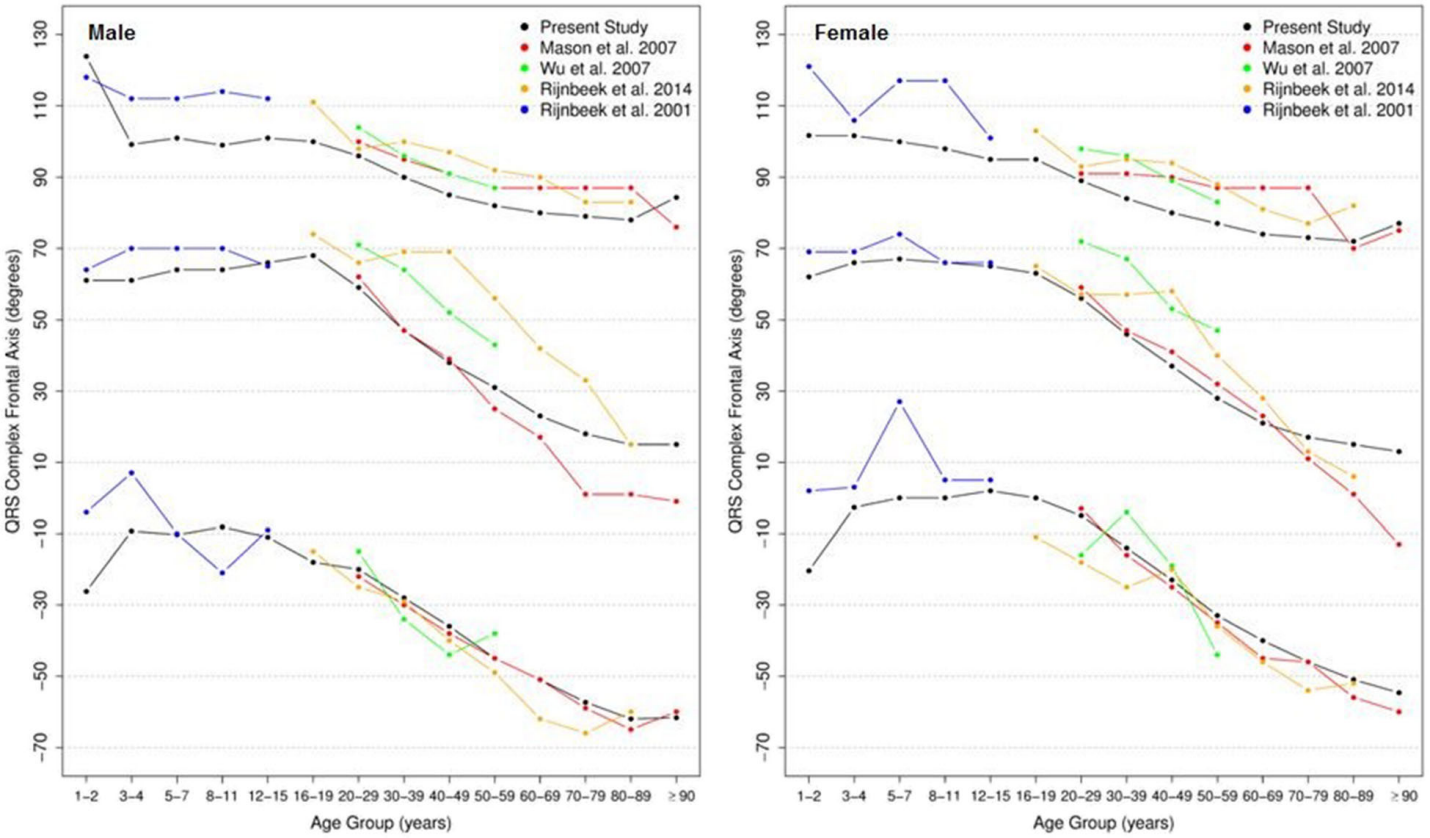
Comparison of lower, median and upper normal limits for QRS-wave frontal axis of different studies according age groups and sex

**Fig. 11:**
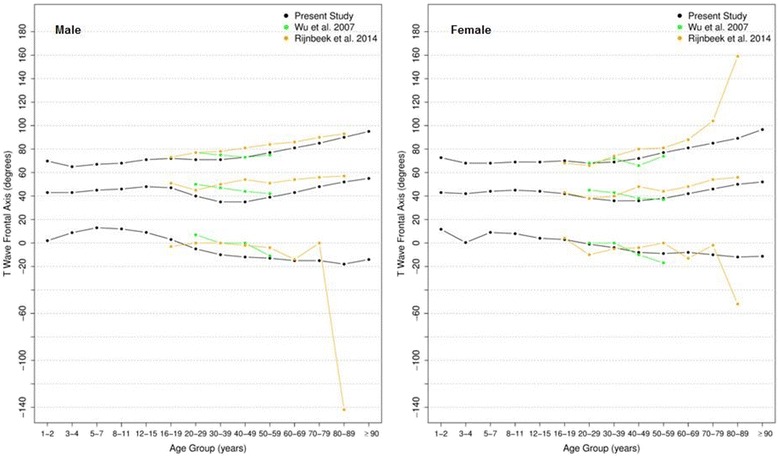
Comparison of lower, median and upper normal limits for T-wave frontal axis of different studies according age groups and sex

Figures 9, 10 and 11 compares our results to the others. The median P frontal axis was quite similar for all ages and sexes to that of Rijnbeek [12, 26], although the percentiles 2 and 98 varied a little for males and females younger than 4 years and older than 80 years.

Although there were similar values for P frontal axis, Rijnbeek’s [12, 26] and Wu’s [7] studies showed larger differences for the QRS and T frontal axes, and in these studies, the axes were seen to be orientated more inferiorly than ours. This difference happened in both sexes, but was more important for males than females. For the QRS frontal axis, our values were more comparable to those of Mason [13] than the others.

#### Overall P and QRS duration

**Fig. 12:**
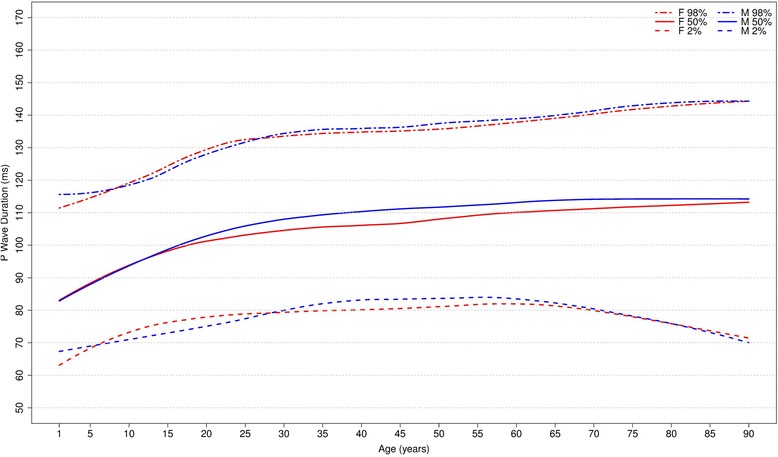
Percentiles 2nd, 50th and 98th for overall P duration according age and sex

Figure 12 shows our results for the normal limits of the overall P duration, which is slightly greater for men than for women. The median value increases from about 85 ms for both sexes in childhood to 114 ms in men and 112 ms in women after 90 years-old.

**Fig. 13:**
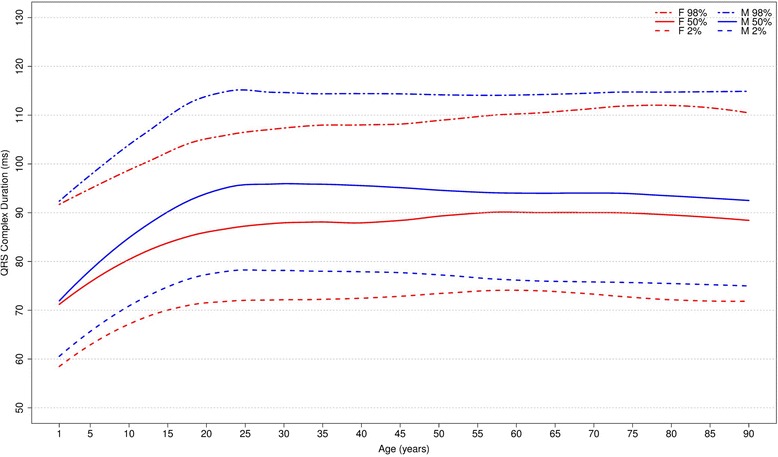
Percentiles 2nd, 50th and 98th for overall QRS duration according age and sex

Figure 13 shows our results for the overall QRS duration. The median duration has an important rise from the early phase of life until early adulthood in both sexes. Then, the median QRS duration remains stable around 92-96 ms and 88–90 ms for men and women, respectively. The median P and QRS durations are higher for men in almost all age groups.

**Fig. 14:**
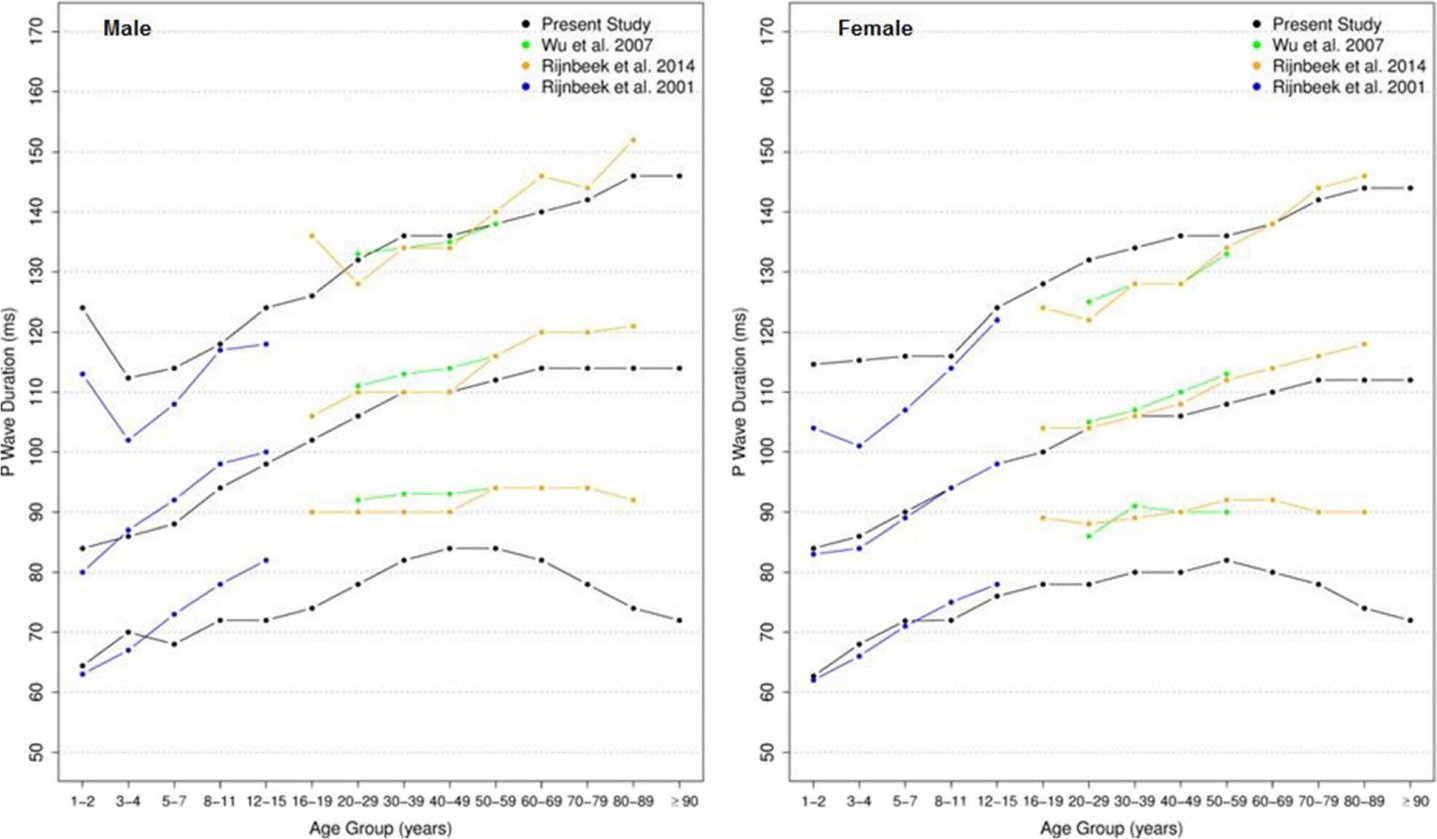
Comparison of lower, median and upper normal limits for overall P duration of different studies according age groups and sex

**Fig. 15:**
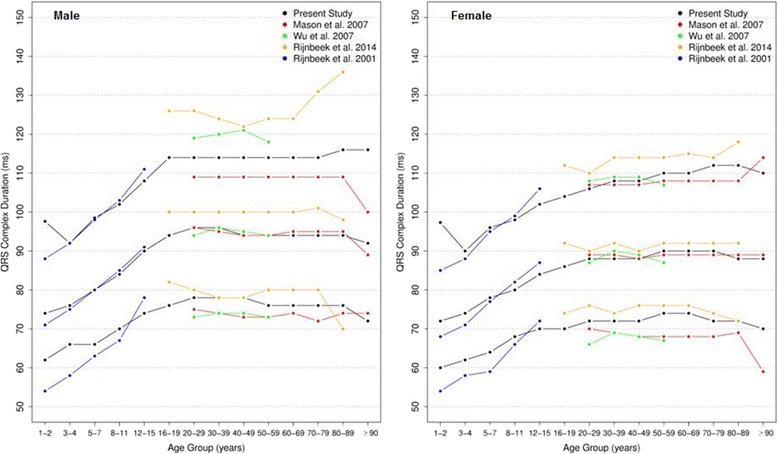
Comparison of lower, median and upper normal limits for overall QRS duration of different studies according age groups and sex

Figures 14 and 15 compares our results to the other studies. The median values of overall P duration for both sexes and all ages were similar to those of to Rijnbeek [12, 26] and Wu [7]. However, some differences were observed for the 2nd percentile from older than 60 years and for the 98th percentile from 1 to 4 years, in males and females.

The comparison of overall QRS duration showed similar results for the first, second and fifth percentiles in all studies, for both sexes and all age groups. However, percentiles 98 and 99 appeared to be slightly different for men older than 90 years in Mason’s study and also the percentile 98 for men from 16 to 29 years and 70–89 years in Rijnbeek’s [12, 26] study. For women, a small difference was seen for the 2nd percentile in subjects older than 90 years in Mason’s [13] study and for the 98th percentile from 1 to 2 years in comparison with Rijnbeek’s [12, 26] study.

#### Overall PR intervals

**Fig. 16:**
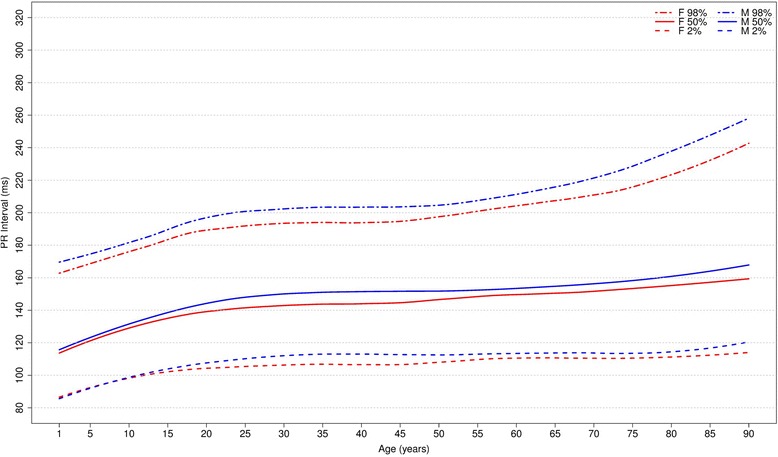
Percentiles 2nd, 50th and 98th for overall PR interval according age and sex

Figure 16 shows our results for the normal limits of the overall PR intervals. The median value is slightly higher for men in all age groups and increases with age in both sexes. In age groups 30–39 and 80–89 years, the medians are, respectively, 150 ms and 164 ms for men and 144 ms and 156 ms for women.

**Fig. 17:**
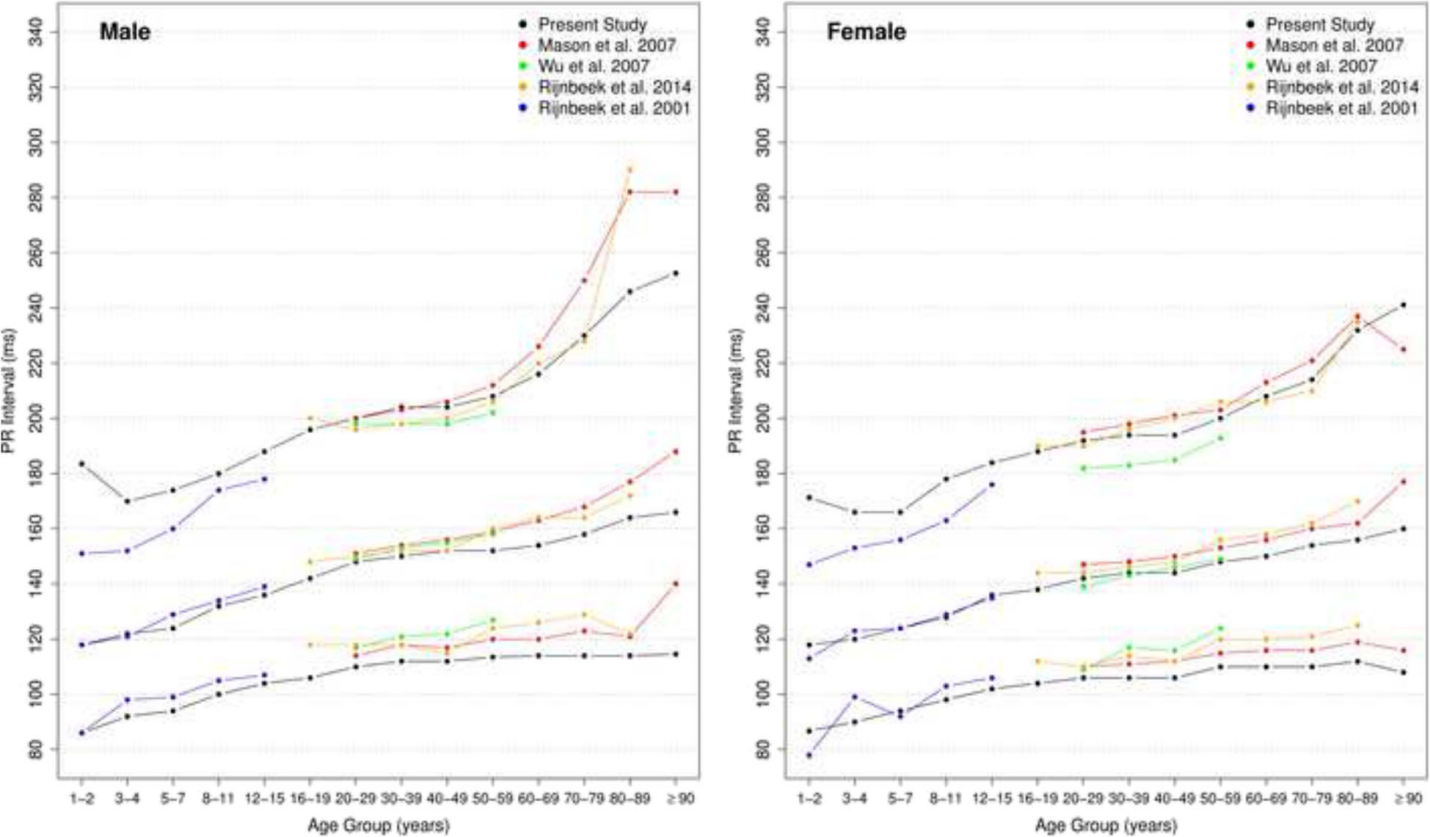
Comparison of lower, median and upper normal limits for overall PR interval of different studies according age groups and sex

The median overall PR intervals were compared in all studies (Fig. 17) and they appeared to have similar values. However, some discrepant values were seen in extreme age groups in both sexes. Some discrepancies were seen in the 2nd percentile in men older than 90 years in Mason’s [13] study and in the 98th percentile for children from 1 to 7 years and men older than 80 years in Rijnbeek’s [12, 26] studies and in men older than 70 years and women older than 90 years old in Mason’s [13] study.

#### QT interval

**Fig. 18:**
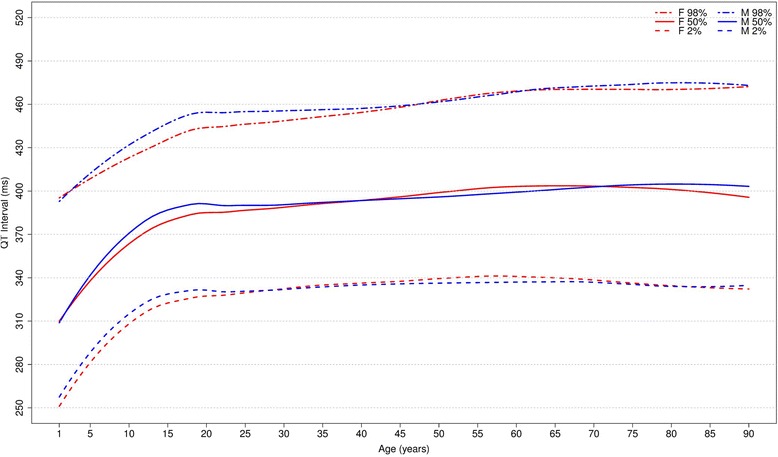
Percentiles 2nd, 50th and 98th for overall QT interval according age and sex

Figure 18 shows our results for the normal limits of the QT interval. It increases progressively from 1 to 89 years old in men and from 1 to 79 in women and reduces a little in the last phase of life. The values are quite similar in both sexes for each age group.

**Fig. 19:**
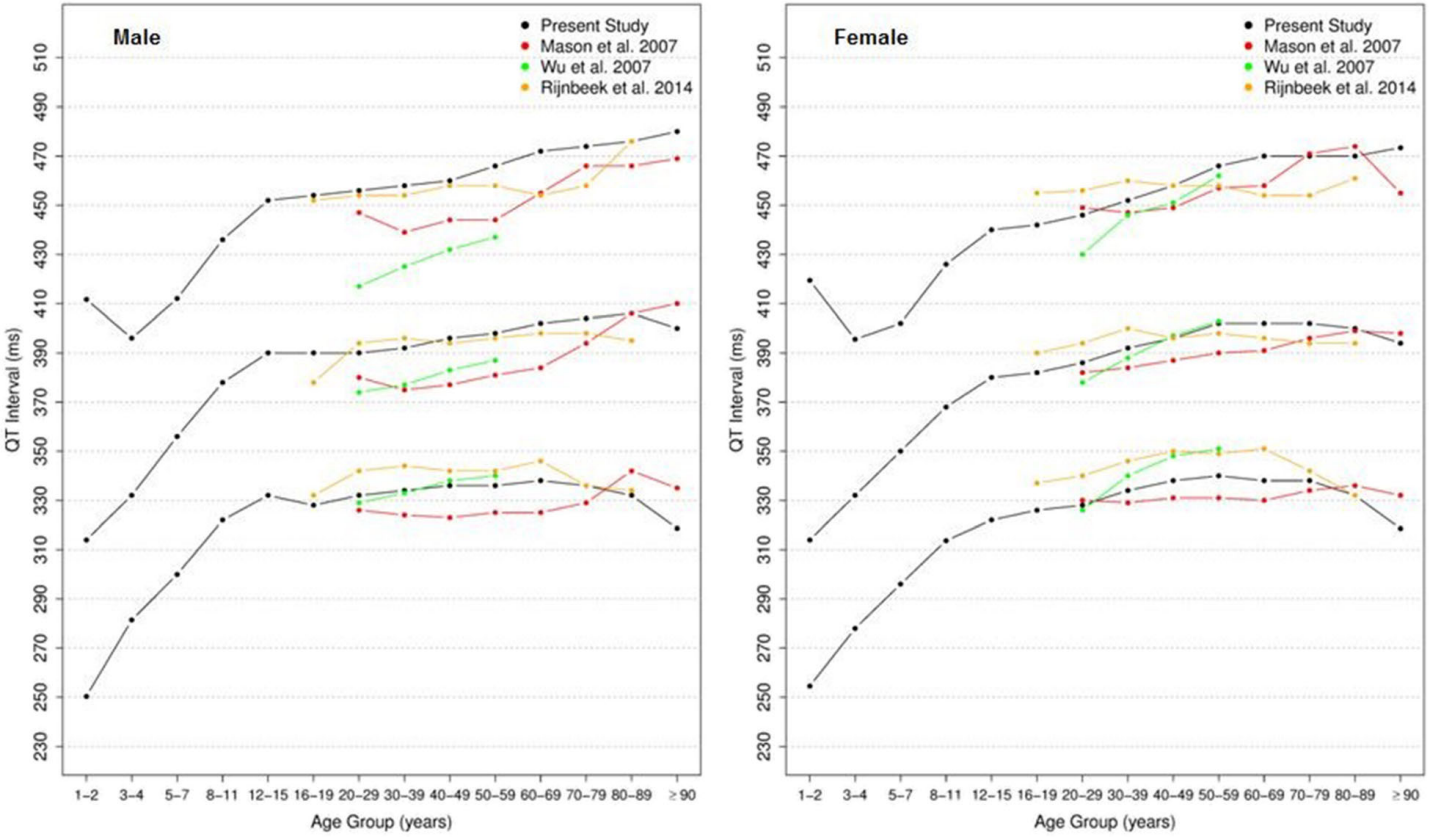
Comparison of lower, median and upper normal limits for overall QT interval of different studies according age groups and sex

Figure 19 compares it to the other studies. For men, the values of QT interval in our study were quite similar to those of Rijnbeek [12], while the values from Mason [13] and Wu [7] were longer than ours. For women, all the studies had similar values, except the percentiles 1, 2, 98 and 99 for subjects older than 90 years in Mason’s [13] study, which appeared to have a wide range.

#### QTc Hodges, Bazett, Fridericia, Framingham [28–31]

**Fig. 20:**
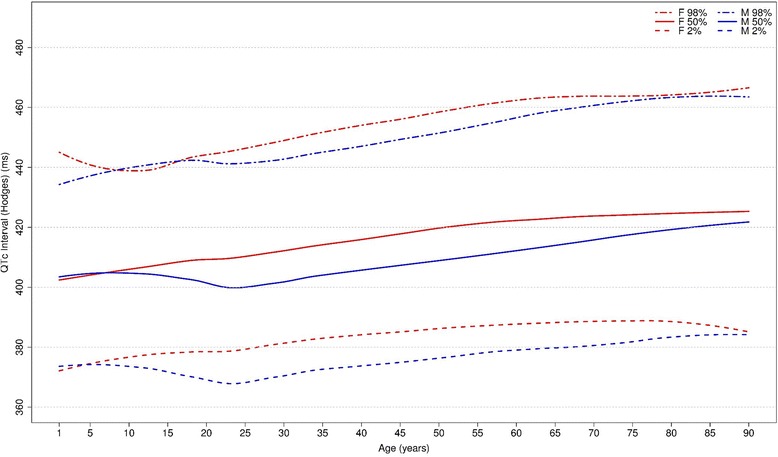
Percentiles 2nd, 50th and 98th for QTc Hodges according age and sex

**Fig. 21:**
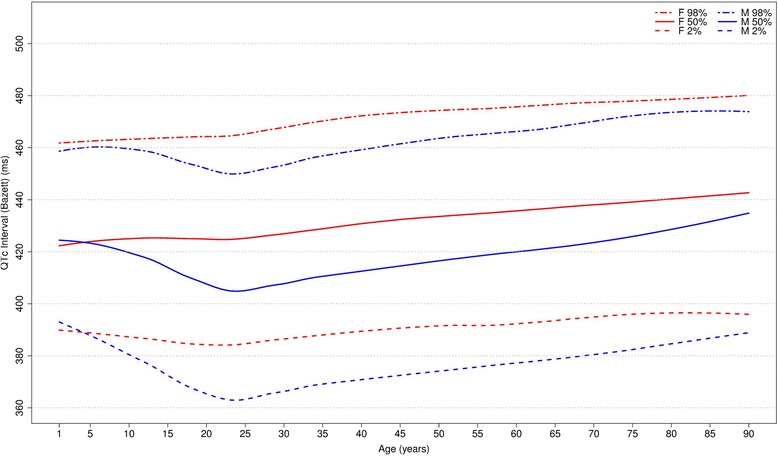
Percentiles 2nd, 50th and 98th for QTc Bazett according age and sex

**Fig. 22:**
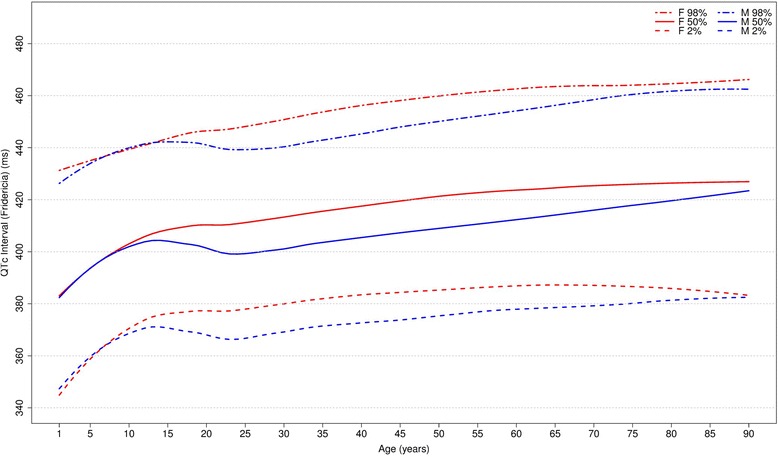
Percentiles 2nd, 50th and 98th for QTc Fridericia according age and sex

**Fig. 23:**
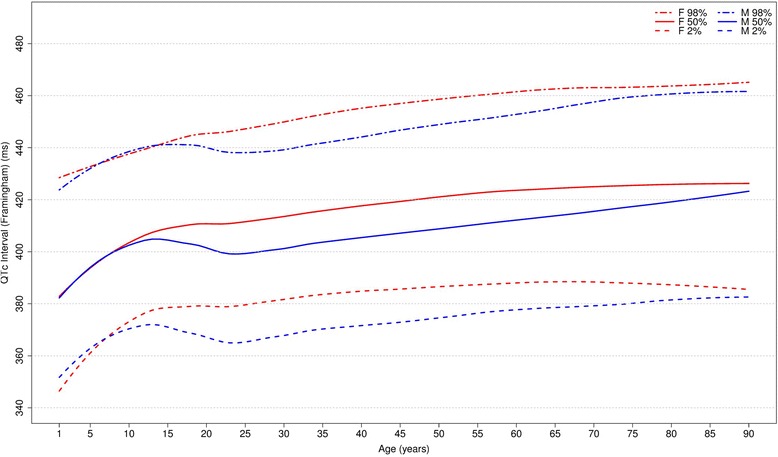
Percentiles 2nd, 50th and 98th for QTc Framingham according age and sex

Figures 20, 21, 22 and 23 show our normal limits of the QTc using four different methods of correction. Women have higher medians, upper and lower limits than men in almost all age groups. The Bazett method showed higher values in both sexes for all ages in comparison to the other methods, whereas Hodges, Fridericia and Framingham showed values similar to each other. In the Bazett method, the median and 2nd and 98th percentiles have a fast decrease during childhood and adolescence, followed by a progressive increase until they reach a median 421 ms and 437 ms for men and women, respectively, from 60 to 69 years-old. Hodges, Fridericia and Framingham have similar behaviors: their median and percentiles initially describe an upward concavity during childhood. After adolescence, they have a linear increase with aging. The median for men and women from 60 to 69 years-old is, respectively, 421 ms and 437 ms (Bazett), 414 ms and 423 ms (Hodges), 414 ms and 425 ms (Fridericia) and 414 ms and 424 ms (Framingham).

**Fig. 24:**
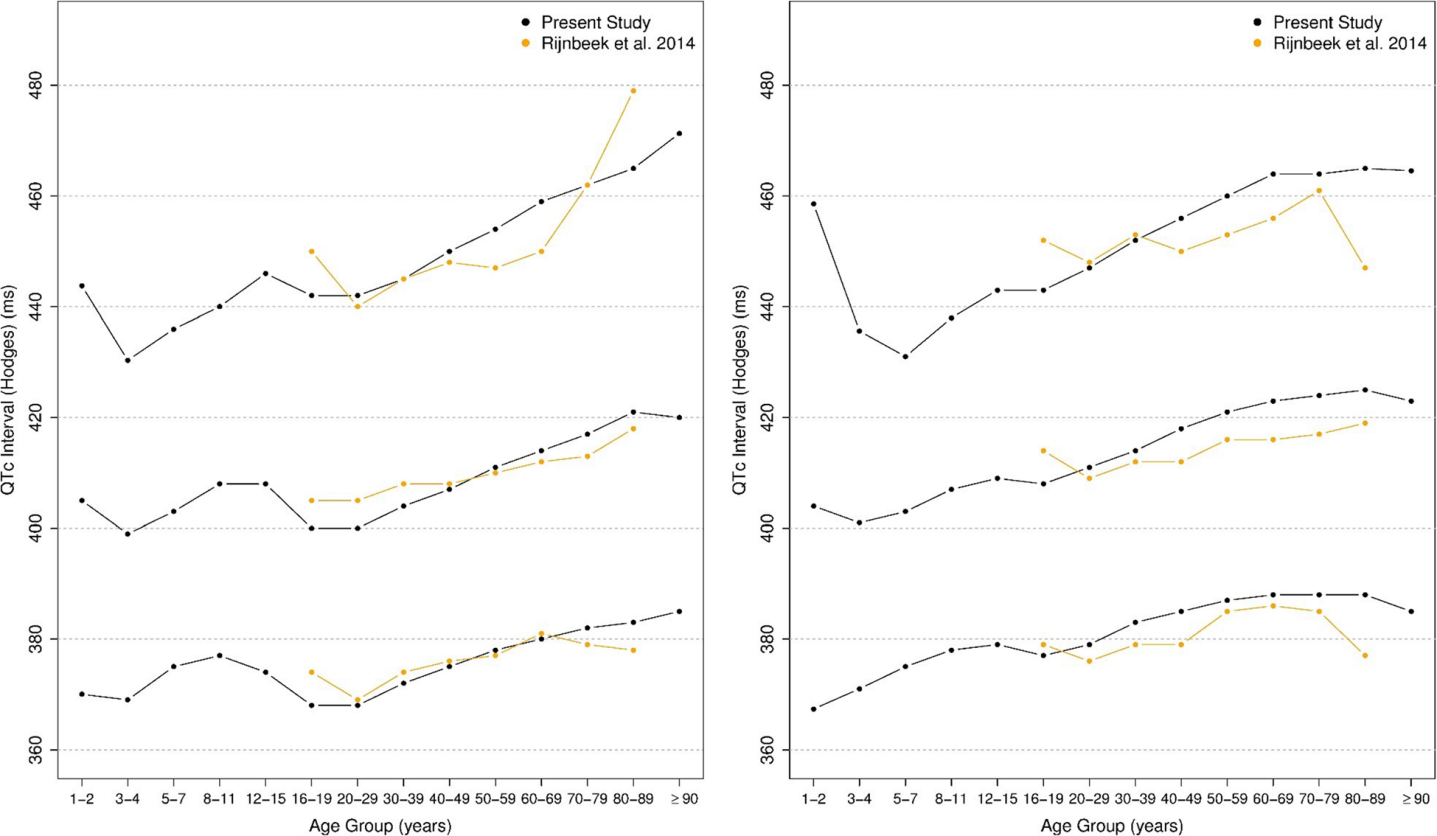
Comparison of lower, median and upper normal limits for QTc Hodges of different studies according age groups and sex

**Fig. 25:**
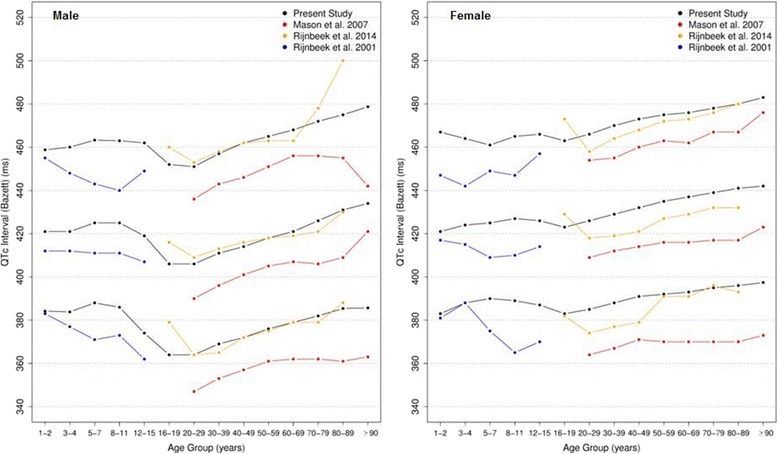
Comparison of lower, median and upper normal limits for QTc Bazett of different studies according age groups and sex

**Fig. 26:**
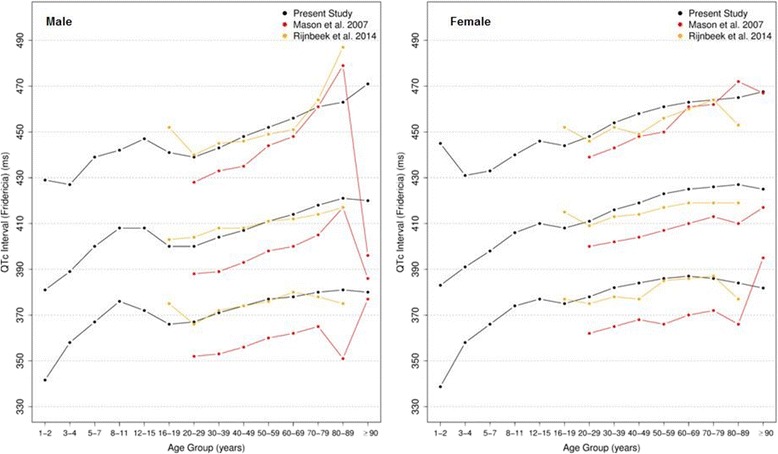
Comparison of lower, median and upper normal limits for QTc Fridericia of different studies according age groups and sex

**Fig. 27:**
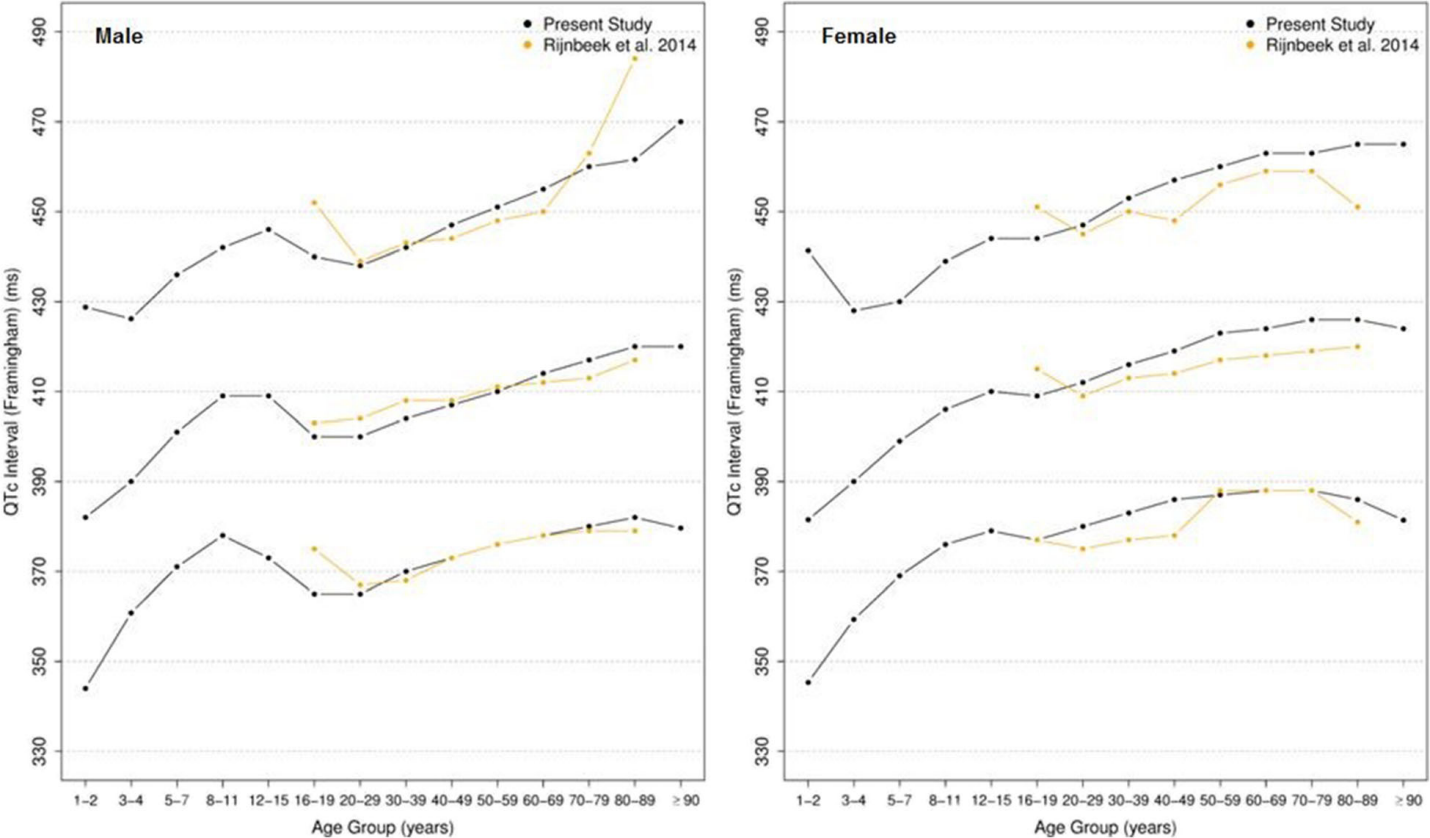
Comparison of lower, median and upper normal limits for QTc Framingham of different studies according age groups and sex

The corrected QT intervals were compared to other studies as shown in Figs. 24, 25, 26 and 27. The QTc Hodges and QTc Framingham were very similar in our study and in the study of Rijnbeek [12], and the only large difference occurred in the 98th percentile for men and the 2nd and 98th percentiles for women from 80 to 90 years old. QTc Bazett and QTc Fridericia were compared to the results of Mason [13] and Rijnbeek [12, 26]. Overall, the results of Rijnbeek’s [7, 12, 26] study were quite similar to ours, whereas Mason’s [13] study had higher values than ours, for all percentiles, irrespective of age and sex.

## Discussion

We studied the normal limits of the ECG in almost 500,000 pediatric and adult patients from primary care centers in the state of Minas Gerais Brazil, using automated analysis by an internationally recognized computer program [5]. To our knowledge, this is the largest study that has derived ECG reference values and it is the first performed exclusively in Latinos. Other authors have undertaken similar studies in different populations, but using smaller sample sizes, without including all the variables and age groups that we did [6–13].

Our population was large enough to permit very reliable measures from all age groups for both sexes, even at the extremes of age. In addition, the large sample size was important to create graphs with minimal smoothing, which would not be possible in smaller populations. For example, we created 14 age groups with an average of 34,715 patients in each group (ranging from 475 to 92,356) and included more than 39,000 patients in their seventies or older. We divided the patients into age groups similar to other studies and chose percentiles that had previously been adopted to allow comparison of reference values. Although the Brazilian population has a high level of miscegenation, we did not split up this population according to race.

Overall, our results were similar to those of other studies. Some larger differences were observed in extreme ages and extreme percentiles, which might have occurred mainly because of the small number of patients of extreme age in other studies but also due to differences in study population (race; inclusion or exclusion criteria).

Our study included 39,098 patients older than 70 years old while the studies of Mason [13] and Rijnbeek [12] included 5139 and 942 patients, respectively, for the same age group. In addition, for children, we included 1982 patients from 1 to 4 years old, while Rijnbeek [26] studied only 363. As we had a considerably greater number of patients in extreme age groups, we consider that for young children and seniors our results might be more stable than the data from other studies.

Most previous studies recruited healthy volunteers from the community with a normal cardiovascular system and included a clinical examination in order to exclude those with evidence of any illness likely to affect the cardiovascular system and possibly the ECG. As we used a database that contained each study participant’s ECG and clinical information based on a patient’s self-report and medical record, we excluded individuals with known clinical conditions likely to affect the cardiovascular system, those on any kind of drugs and/or with frankly abnormal Minnesota Codes.. We opted to maintain the 6454 (1.3%) patients with premature beats (ventricular, supraventricular or combined) since it might happened in the “normal” population [8]. In summary, we have excluded patients with conditions that might affect the cardiovascular system and we accepted that the remaining population was an “apparently healthy population”. However, since the fulfillment of patient data was undertaken by professionals in primary care, some diagnostic errors or underreporting of latent illness may have occurred, and this is one of our study limitations. On the other hand, the extremely large number of study participants should ensure that any measurements outliers due to such shortcomings will not have significantly affected the normal 2nd to 98th percentile normal ranges.

In broad terms, our results for QTc are very similar to the detailed paper by Luo et al. [33] who showed that QT corrected by Bazett was generally out of line with QTc corrected by the Hodges, Fridericia and Framingham methods.

Results of PR interval measurements are also of considerable interest. An upper limit of normal of 220 ms is often suggested but our data show two points. First of all, the large sample size indicates that males have a longer PR interval than females by around 20 ms which may be accounted for by heart size and secondly the upper limit of normal increases rapidly above 70 years of age from 220 ms to 260 ms at age 90 in men and from 200 ms to 240 ms in women.

Once all the measures were made using computers, the reference values might be valid for computerized analyses and not manual measurements on paper. One other factor that has to be acknowledged is that the automated approach itself is subject to what might be termed inter-program variation analogous to differences in measurements between two or more cardiologists, i.e. inter observer variation. In a comparison of 4 major algorithms using the same ECGs, it was shown that the mean QT interval varied by 8 ms in a group of 200 normal subjects [21]. Although the sample size was small, the inference is clear that some variation in QT measurements between studies is due to the different algorithms used to derive the results. On this subject, it should be noted that the other four studies used different hardware and software as well as different analysis programs, which might be a confounding factor for accurately compare to our data. Also, their sample selection methods and populations sampled were different with the potential to affect direct measurement comparisons.

Although the ECG intervals are influenced by sex and age, simpler reference values like normal heart rate 60-100 bpm or QRS duration <100 ms for all ages and both sexes, are commonly used in primary care centers, and even in medical education. We recommend that those old normal ranges should be discarded and replaced by age and sex-specific values. In that respect, we consider that graphs and tables with the percentiles may help general practitioners undertaking ECG analysis.

Finally, this paper has concentrated on the comparison of ECG interval and axes measurements in different populations. In due course, it is hoped to produce a detailed comparison for the major ECG wave component amplitudes.

## Conclusion

This study contributes to the knowledge of the reference values in Latinos. Although we have seen small differences in comparison to studies in other populations, reference values for electrocardiogram intervals and axes in Latinos are in general comparable to those obtained in those other populations.

## Abbreviations

ECG: Electrocardiogram
MC: Minnesota code
QTc: Corrected QT
QTi: QT index
TNMG: Telehealth Network of Minas Gerais
Uni-G: The University of Glasgow ECG Analysis Program
